# Practice-Level Documentation of Alcohol-Related Problems in Primary Care

**DOI:** 10.1001/jamanetworkopen.2023.38224

**Published:** 2023-10-19

**Authors:** Elizabeth Needham Waddell, George S. Leibowitz, Levi N. Bonnell, Gail L. Rose, Mark McGovern, Benjamin Littenberg

**Affiliations:** 1 OHSU-PSU School of Public Health & OHSU School of Medicine, Oregon Health & Science University, Portland; 2 Stony Brook University School of Social Welfare, Stony Brook, New York; 3 Larner College of Medicine, University of Vermont, Burlington; 4 Stanford University School of Medicine, Stanford, California

## Abstract

**Importance:**

Rates of alcohol-associated deaths increased over the past 20 years, markedly between 2019 and 2020. The highest rates are among individuals aged 55 to 64 years, primarily attributable to alcoholic liver disease and psychiatric disorders due to use of alcohol. This study investigates potential geographic disparities in documentation of alcohol-related problems in primary care electronic health records, which could lead to undertreatment of alcohol use disorder.

**Objective:**

To identify disparities in documentation of alcohol-related problems by practice-level social deprivation.

**Design, Setting, and Participants:**

A cross-sectional study using secondary data from the Integrating Behavioral Health and Primary Care clinical trial (September 21, 2017, to January 8, 2021) was performed. A national sample of 44 primary care practices with co-located behavioral health services was included in the analysis. Patients with 2 primary care visits within 2 years and at least 1 chronic medical condition and 1 behavioral health condition or at least 3 chronic medical conditions were included.

**Exposure:**

The primary exposure was practice-level Social Deprivation Index (SDI), a composite measure based on county income, educational level, employment, housing, single-parent households, and access to transportation (scores range from 0 to 100; 0 indicates affluent counties and 100 indicates disadvantaged counties).

**Main Outcomes and Measures:**

Documentation of an alcohol-related problem in the electronic health record was determined by *International Classification of Diseases, 9th Revision, Clinical Modification* and *International Statistical Classification of Diseases and Related Health Problems, Tenth Revision, Clinical Modification* codes or use of medications for alcohol use disorder in past 2 years. Multivariable models adjusted for alcohol consumption, screening for a substance use disorder, urban residence, age, sex, race and ethnicity, income, educational level, and number of chronic health conditions.

**Results:**

A total of 3105 participants (mean [SD] age, 63.7 [13.0] years; 64.1% female; 11.5% Black, 7.0% Hispanic, 76.7% White, and 11.9% other race or chose not to disclose; 47.8% household income <$30 000; and 80.7% urban residence). Participants had a mean (SD) of 4.0 (1.7) chronic conditions, 9.1% reported higher-risk alcohol consumption, 4% screened positive for substance use disorder, and 6% had a documented alcohol-related problem in the electronic health record. Mean (SD) practice-level SDI score was 45.1 (20.9). In analyses adjusted for individual-level alcohol use, demographic characteristics, and health status, practice-level SDI was inversely associated with the odds of documentation (odds ratio for each 10-unit increase in SDI, 0.89; 95% CI, 0.80 to 0.99; *P* = .03).

**Conclusions and Relevance:**

In this study, higher practice-level SDI was associated with lower odds of documentation of alcohol-related problems, after adjusting for individual-level covariates. These findings reinforce the need to address primary care practice-level barriers to diagnosis and documentation of alcohol-related problems. Practices located in high need areas may require more specialized training, resources, and practical evidence-based tools that are useful in settings where time is especially limited and patients are complex.

## Key Points

**Question:**

Within primary care, are there disparities in documentation of alcohol-related problems related to practice location?

**Findings:**

In this cross-sectional study including 3105 patients, primary care practices located in counties with greater social deprivation, as indicated by a standardized Social Deprivation Index, were less likely to document alcohol-related problems in the electronic health record. These findings were observed after adjusting for individual-level alcohol use, demographic characteristics, and health status.

**Meaning:**

The findings of this study suggest that practices located in counties with high levels of social deprivation may require specialized training, resources, and practical evidence-based tools to improve documentation of alcohol-related problems in contexts in which time is especially limited and patients are complex.

## Teaser

This cross-sectional study investigates whether disparities exist in documentation of alcohol-related problems by practice-level social deprivation.

## Introduction

### Background/Rationale

Rates of alcohol-associated deaths in the US increased 26% between 2019 and 2020, from 10.4 deaths per 100 000 standard population to 13.1 deaths per 100 000 standard population.^1^ The highest rates were in the 55- to 64-year age group and attributable primarily to increases in alcoholic liver disease and comorbid psychiatric disorders related to alcohol use.^1^ Medicare data from 2007 to 2014 indicate a 19% increase in hospitalization rates associated with alcohol use disorder (AUD), from 485 to 579 per million adults aged 65 years and older, with even higher rates for Black older adults.^2^

Previous studies examining documentation of AUD in primary care suggest substantial gaps in screening, diagnosis, and treatment of alcohol-related problems, even in systems with population-based screening.^3,4^ Research assessing disparities in screening for AUDs in the US and the UK have attributed low rates of documentation to practice-level, clinician-level, or patient-level characteristics.^5,6,7,8,9,10,11,12,13,14^ Prior research rarely explores the association between poor documentation and geographic indicators of social deprivation. Clinical factors related to incomplete documentation include poor follow-up, lack of decision support built into electronic health records (EHRs) to facilitate documentation and billing,^5^ lack of clinical knowledge or training, and practice-level factors including time, space, and access to addiction specialists.^6^ Perceived stigma on the part of both patients and clinicians is known to be associated with screening and diagnosis of alcohol-related problems in primary care, resulting in both reduced patient disclosure and reduced screening and documentation by clinicians.^6,7,8,9,10,11,12^ The 2014 National Survey on Drug Use and Health suggests that men, racial and ethnic minority individuals, and uninsured patients are less likely to be asked about their alcohol use by a health care professional,^13^ while the National Ambulatory Medical Care Survey data showed no association between sex or race and ethnicity on screening.^14^

Few studies have compared self-report data with EHRs to assess documentation of alcohol use disorder, positive screenings, or alcohol-related problems. Williams et al^15^ compared routine screenings for unhealthy alcohol use with Veteran’s Administration medical records and found that 25% of patients with positive screens had documented alcohol or substance use disorders in their medical records. Abidi et al^16^ assessed agreement between patient self-report and clinical documentation of alcohol abuse in the Netherlands. Among 261 patients with positive Alcohol Use Disorders Identification Test-C screens, only 3 had a documented alcohol problem. Williams et al^17^ found that survey-based use disorder rates exceeded documented diagnoses rates across demographic subgroups.

The epidemiologic composition of AUD is complex, and it is challenging to evaluate the combined associations among stigma, race and ethnicity, gender, educational level, social and economic status, and diagnosis and treatment for AUD. Internationally, AUD is diagnosed more often in high-income countries, while individuals within lower socioeconomic groups experience greater harm from alcohol.^18^ Recognizing that disparities in documentation of alcohol-related problems may present differently at the practice and the person level, this study specifically assesses the association between county socioeconomic status at practice level and odds of documenting alcohol-related problems in the EHR. A county’s socioeconomic status may directly impact a practice’s capacity for comprehensive patient care. Practice-level barriers to diagnosis and documentation of alcohol-related disorders, as well as other behavioral health conditions, can be tied to local public and private insurance payment rates, workforce shortages, larger patient panel size associated with shortages of primary care within a county, and resources for training all levels of staff.

The present study uses patient survey and corresponding EHR data from the Integrating Behavioral Health and Primary Care (IBH-PC) clinical trial^19,20^ to estimate the association between the practice-level Social Deprivation Index (SDI),^21^ a standardized measure of socioeconomic deprivation within the practice’s county, and clinically documented alcohol-related problems in the EHR.

The objective of this cross-sectional study was to identify disparities in documentation of alcohol-related problems associated with a practice-level SDI score,^21^ controlling for patient-level alcohol use, sociodemographic characteristics, and health status. The practice-level SDI score indicates the level of social deprivation within the practice’s county.

## Methods

### Study Design

This cross-sectional study used baseline patient survey and EHR data from the IBH-PC multicenter pragmatic trial.^19^ Data were collected between September 21, 2017, and January 18, 2021, from 3105 adults who received care from 44 primary care practices with colocated behavioral health services. The University of Vermont Institutional Review Board approved this study. The patients provided informed consent and received financial compensation. This report follows the Strengthening the Reporting of Observational Studies in Epidemiology (STROBE) reporting guideline for cross-sectional studies.

### Setting

The IBH-PC trial took place in 44 primary care clinics across the US and represented varied geographic regions, patient population sizes, population densities, and specialties. Practices included community health centers, federally qualified health centers, nonprofit and for-profit organizations, and resident training sites. Hospitals and health centers, academic clinics, and private practices were included.^19^

### Participants

Eligible participants were current primary care patients with at least 1 chronic medical and 1 behavioral health condition or at least 3 chronic medical conditions. The presence of each qualifying condition was determined by EHR data. Medical conditions included arthritis, obstructive lung disease, nongestational diabetes, and heart disease. Behavioral health conditions included mood disorder, chronic pain, insomnia, irritable bowel syndrome, and substance misuse.^19^

### Variables and Data Sources

The primary outcome was the documentation of an alcohol-related problem in the EHR as determined by *International Classification of Diseases, 9th Revision, Clinical Modification* and *International Statistical Classification of Diseases and Related Health Problems, Tenth Revision, Clinical Modification* codes (Table 1) or use of medications for AUD (acamprosate, disulfiram, or naltrexone) during the 2 years before study entry.

**Table 1.** *ICD* Codes Used to Document Alcohol Issues

*ICD* code	Description
*ICD-9-CM*	
291.4	Idiosyncratic alcohol intoxication
303.01	Acute alcoholic intoxication in alcoholism, continuous
303.02	Acute alcoholic intoxication in alcoholism, episodic
303.03	Acute alcoholic intoxication in alcoholism, in remission
303.91	Other and unspecified alcohol dependence, continuous
303.92	Other and unspecified alcohol dependence, episodic
305.01	Alcohol abuse, continuous
305.02	Alcohol abuse, episodic
305.03	Alcohol abuse, in remission
790.3	Excessive blood level of alcohol
980	Toxic effect of alcohol
*ICD-10-CM*	
F10.1	Alcohol abuse
F10.12	Alcohol abuse with intoxication
F10.121	Alcohol abuse with intoxication delirium
F10.14	Alcohol abuse with alcohol-induced mood disorder
F10.150	Alcohol abuse with alcohol-induced psychotic disorder with delusions
F10.151	Alcohol abuse with alcohol-induced psychotic disorder with hallucinations
F10.180	Alcohol abuse with alcohol-induced anxiety disorder
F10.180	Alcohol abuse with alcohol-induced anxiety disorder
F10.180	Alcohol abuse with alcohol-induced anxiety disorder
F10.181	Alcohol abuse with alcohol-induced sexual dysfunction
F10.182	Alcohol abuse with alcohol-induced sleep disorder
F10.188	Alcohol abuse with other alcohol-induced disorder
F10.2	Alcohol dependence
F10.21	Alcohol dependence, in remission
F10.22	Alcohol dependence with intoxication
F10.23	Alcohol dependence with withdrawal
F10.231	Alcohol dependence with withdrawal delirium
F10.25	Alcohol dependence with alcohol-induced psychotic disorder
F10.26	Alcohol dependence with alcohol-induced persisting amnestic disorder
F10.27	Alcohol dependence with alcohol-induced persisting dementia
F10.280	Alcohol dependence with alcohol-induced anxiety disorder
F10.9	Alcohol use, unspecified
G62.1	Alcoholic polyneuropathy
I42.6	Alcoholic cardiomyopathy
K29.20	Alcoholic gastritis without bleeding
K29.21	Alcoholic gastritis with bleeding
K70.0	Alcoholic fatty liver
K70.10	Alcoholic hepatitis without ascites
K70.30	Alcoholic cirrhosis of liver without ascites
K70.9	Alcoholic liver disease, unspecified
R78.0	Finding of alcohol in blood

Abbreviations: *ICD-9-CM*, *International Classification of Diseases, 9th Revision, Clinical Modification*; *ICD-10-CM*, *International Statistical Classification of Diseases and Related Health Problems, Tenth Revision, Clinical Modification*.

The primary risk factor was practice-level SDI. The SDI score assigned to each practice is a composite measure of county deprivation based on income, educational level, employment, housing, single-parent households, and access to transportation. The SDI is a continuous scale with scores ranging from 0 to 100, where 0 indicates affluent counties and 100 indicates disadvantaged counties.^21^

The model adjusts for individual-level risk for an alcohol-related problem using 2 indicators: (1) self-reported alcohol use and (2) positive screen for substance use disorder. Higher risk, lower risk, and no alcohol consumption indicators were derived from self-report patient surveys collected for the IBH-PC trial.^19^ Alcohol units consumed per month and per day were obtained from the 2-item Self-Report Habit Index–Alcohol, which has been shown to have excellent reliability.^22^ Risk of SUD was assessed using the 5-item Substance Disorders subscale of the Global Assessment of Individual Needs–Short Screener (GAIN-SS), a biopsychosocial screener for individuals presenting with substance use and mental health concerns.^23^ The GAIN-SS Substance Use subscale has been shown to have acceptable reliability (Cronbach α ranging from 0.77 to 0.84). The GAIN-SS includes 5 items indicating use or consequences of use of alcohol or drugs. For each item there is a score of 0 (unlikely), 1 to 2 (moderate), and 3+ (high), indicating the likelihood of a substance use disorder diagnosis.^24^

As do the National Institute on Alcohol and Alcoholism^25^ and the Centers for Disease Control and Prevention,^26^ we incorporated indicators of both binge drinking and average daily drinks into our indicator of higher risk drinking, using the data that were available. The specific questions asked on the item Self-Report Habit Index–Alcohol were, “During the past 30 days, on how many days did you drink one or more alcoholic beverages?” and “On the days that you drank during the 30 days, how many drinks did you usually have each day? (count as a drink a can or bottle of beer; a wine cooler or glass of wine, champagne or sherry; a shot of liquor, or a mixed drink or cocktail).” From these items, we calculated a 3-level categorical variable to indicate level of alcohol use: 0, no use; 1, lower risk; and 2, higher risk. Participants were assigned to the higher risk category if they met 1 of the following qualifying conditions: (1) more than 4 drinks in any given day, (2) more than 30-monthly drinks for women, (3) more than 30-monthly drinks for men older than 65 years, and (4) more than 60-monthly drinks for men.

Individual demographic variables are from self-report patient surveys collected for the IBH-PC trial.^19^ These included age in years, sex (male and female), race (Black, White, and other), ethnicity (Hispanic and non-Hispanic), educational level (college degree or more and less than college degree), number of chronic conditions, and urban-rural status of the practice’s census tract level as defined by the Rural-Urban Commuting Area codes.^27^ Race and ethnicity, known social determinants of health, were included in the analysis as they are theoretical predictors of both alcohol use and likelihood of screening for and disclosure of alcohol-related problems.

The 3 continuous variables (age, SDI score, and number of chronic conditions) were checked for nonlinear relationships with documentation of alcohol using the Box-Tidwell test as well as graphically using restricted cubic splines in univariate and the multivariate models. The SDI score and number of chronic conditions were linearly related to the outcome. However, age showed an inverse U relationship, with the most prominent relationship being older than 70 years (those <50 and >70 years were less likely to be documented). Based on these findings, both age and age squared were included in the models.

### Statistical Analysis

We summarized demographic and health characteristics and practice SDI by self-reported alcohol use. Unadjusted mixed-effects logistic regression models were used to compare practice SDI, demographic, and health characteristics with documented alcohol use in the EHR, with a random intercept for practice. Multivariable mixed-effects logistic regression was used to compare practice SDI with documented alcohol use in the EHR, with a random intercept for practice. Analyses were performed in Stata, version 16.1 (StataCorp LP). With 2-sided, unpaired testing, the threshold for statistical significance was *P* < .05. Adjustment for multiple comparisons was not required to assess the single primary outcome between SDI and documentation of an alcohol-related problem.

## Results

### Participants

Table 2 summarizes characteristics of the IBH-PC study population who completed baseline survey assessments describing their alcohol consumption in the past 30 days (N = 3105). Of these individuals, 64.1% were female, 35.6% were male, 11.5% were Black, 7.0% reported Hispanic ethnicity, 76.7% were White, 11.9% reported another race or chose not to disclose, 47.8% had household incomes less than $30 000, and 80.7% lived in urban areas. Participants had a mean (SD) of 4.0 (1.7) chronic conditions, 9.1% reported higher risk drinking, and 4% screened positive for SUD on the GAIN-SS. The mean (SD) SDI score across practices was 45.1 (20.9). Within the higher-risk alcohol use group, 24% of the individuals had a positive GAIN-SS screen, compared with 4% of participants in the lower-risk group and 1% of the no-use group.

**Table 2.** Characteristics of Study Population Stratified by Self-Reported Alcohol Use In Past 30 Days

Characteristic	Total No. (%)	No. (%)			
		Higher risk (n = 284 [9.1%])	Lower risk (n = 1003 [32.2%])	No use (n = 1818 [58.6%])	Overall (N = 3105 [100%])
Age, mean (SD), y	3105 (100)	66.7 (11.8)	62.1 (13.2)	64.1 (12.9)	63.7 (13.0)
Sex	3105 (100)^a^				
Male		147 (51.9)	390 (38.9)	569 (31.4)	1106 (35.6)
Female		136 (47.9)	610 (61.8)	1245 (68.5)	1991 (64.1)
Race	3099 (99.9)				
Black		18 (6.3)	106 (10.6)	228 (12.6)	352 (11.5)
White		250 (88.1)	800 (79.8)	1329 (73.3)	2329 (76.7)
Other, including not disclosed^b^		16 (5.6)	96 (9.6)	256 (14.1)	368 (11.9)
Hispanic ethncity	3085 (99.4)	18 (6.3)	106 (10.6)	228 (12.6)	352 (11.5)
Low household income (<$30 000)	3042 (98.0)	81 (29.5)	333 (34.1)	1039 (58.8)	1453 (47.8)
College degree	3083 (99.3)	159 (56.6)	582 (58.4)	784 (43.4)	1525 (49.5)
Urban area	3084 (99.3)	224 (78.9)	804 (80.6)	1460 (80.3)	2488 (80.7)
GAIN-SS ≥2 in past mo, mean (SD)^c^	3105 (100)	68 (23.9)	37 (3.7)	18 (1.0)	123 (4.0)
No. chronic conditions in electronic health record, mean (SD)	3105 (100)	3.9 (1.6)	3.8 (1.6)	4.2 (1.7)	4.0 (1.7)
Practice-level SDI score, mean (SD)^d^	3105 (100)	42.5 (20.6)	44.2 (21.0)	45.9 (20.9)	45.1 (20.9)
Documentation of an alcohol-related problem in the EHR	3105 (100)	40 (14.1)	47 (4.7)	95 (5.2)	182 (5.9)

Abbreviations: GAIN-SS, Global Appraisal of Individual Needs–Short Screener; SDI, Social Deprivation Index.

^a^ Includes 8 participants indicating prefer to self-describe or prefer not to say.

^b^ Includes American Indian or Alaskan Native, Asian, Native Hawaiian/Other Pacific Islander, other, and not disclosed.

^c^ The GAIN-SS includes 5 items indicating use or consequences of use of alcohol or drugs. For each item there is a score of 0 (unlikely), 1 to 2 (moderate), and 3+ (high), indicating the likelihood of a substance use disorder diagnosis.

^d^ The SDI is a composite measure of county deprivation based on income, educational level, employment, housing, single-parent households, and access to transportation. SDI is a continuous scale with scores ranging from 0 to 100, where 0 indicates affluent counties and 100 indicates disadvantaged counties.

### Main Results

Six percent of the participants had a documented alcohol-related problem in the EHR. Unadjusted and multivariable regression models to assess the association between practice-level SDI and documentation are summarized in Table 3. For participants with complete data (n = 2944), the median practice-level SDI score was 39 (IQR, 31-63). For ease of interpretation, the 100-point SDI score was recoded to reflect 10-point increments. In the multivariable model, practice-level SDI was inversely associated with odds of documentation. For each 10-unit increase in SDI, there was an associated 11% decrease in the odds of documentation (odds ratio [OR], 0.89; 95% CI, 0.80-0.99; *P* = .03).

**Table 3.** Odds of Documentation of Alcohol-Related Problems in the EHR, Results of Unadjusted and Multivariable Regression Analyses

Variable	Unadjusted regression			Multivariable model (n = 2944)	
	No.	OR (95% CI)	*P* value	OR (95% CI)	*P* value
Practice-level SDI, units of 10	3105	0.96 (0.89-1.03)	.51	0.89 (0.80-0.99)	.03
Self-reported alcohol use in past 30 d					
None	3105	0.99 (0.69-1.44)	.98	0.88 (0.58-1.32)	.52
Lower risk		1 [Reference]	NA	1 [Reference]	NA
Higher risk		3.43 (2.17-5.43)	<.001	3.28 (1.89-5.65)	<.001
GAIN-SS≥2 in past mo	3105	5.35 (3.39-8.69)	<.001	2.11 (1.14-3.90)	<.001
Age	3105	0.98 (0.97-0.99)	<.001	1.11 (0.99-1.24)	.06
Age squared	3105	0.998 (0.997-0.999)	<.001	0.998 (0.997-0.999)	<.001
Sex	3097				
Male		1.84 (1.35-2.50)	<.001	2.04 (1.44-2.99)	<.001
Female		1 [Reference]	NA	1 [Reference]	NA
Race	3099				
Black		0.94 (0.55-1.59)	.81	0.91 (0.52-1.61)	.75
White race		1 [Reference]	NA	1 [Reference]	NA
Other, including not disclosed^a^		0.50 (0.27-0.094)	.03	0.56 (0.27-1.16)	.12
Hispanic ethnicity	3052	0.57 (0.26-1.26)	.16	0.82 (0.34-1.95)	.66
Low household income ($<30 000)	3020	2.37 (1.66-3.37)	<.001	1.84 (1.22-2.78)	.004
College degree	3083	0.50 (0.36-0.71)	<.001	0.86 (0.60-1.26)	.44
Urban area defined by RUCA	3105	0.91 (0.52-1.57)	.73	0.97 (0.89-1.05)	.54
No. chronic conditions in EHR	3105	1.70 (1.54-1.86)	<.001	1.68 (1.52-1.87)	<.001

Abbreviations: EHR, electronic health record; GAIN-SS, Global Appraisal of Individual Needs–Short Screener; NA, not applicable; OR, odds ratio; RUCA, Rural-Urban Commuting Area; SDI, Social Deprivation Index.

^a^ Includes American Indian or Alaskan Native, Asian, Native Hawaiian/Other Pacific Islander, other, and not disclosed.

Higher-risk alcohol use vs lower-risk use (OR, 3.28; 95% CI, 1.89-5.65; *P* < .001), positive GAIN-SS (OR, 2.11; 95% CI, 1.14-3.90; *P* < .001), number of chronic conditions (OR, 1.68, 95% CI, 1.52-1.87; *P* < .001), income lower than $30 000 (OR, 1.84, 95% CI, 1.22-2.78), and male sex (OR, 2.04; 95% CI, 1.44-2.99; *P* < .001) were all associated with increased odds of documentation.

### Other Analyses

Unadjusted mixed-effects logistic regression with a random intercept for practice was used to assess the association between the GAIN-SS and other covariates with the higher-risk alcohol use variable. Results are displayed in eTable 1 in Supplement 1.

Stratifying the multivariable analysis by alcohol use or GAIN-SS categories was not possible due to small cell counts. Instead, a sensitivity analysis was conducted using a parsimonious model excluding GAIN-SS and alcohol use categories from the model. As reported in eTable 2 in Supplement 1, the effect size associated with SDI is similar in the parsimonious model.

## Discussion

### Key Results

Differences in SDI were associated with odds of documentation, after adjusting for alcohol use, demographic characteristics, and health status. Greater social deprivation at the practice level was associated with decreased odds of documentation. At the individual patient level, our adjusted models showed that participants who were male, had household incomes below $30 000, and had higher numbers of chronic conditions were more likely to have alcohol-related problems documented in the EHR.

### Interpretation

The prevalence of AUD is greatest in the world’s wealthiest nations, and self-reported high-risk drinking is typically more prevalent among populations with higher socioeconomic status.^18^ While practice-level SDI was associated with lower odds of documentation, lower socioeconomic status and poorer health at the patient level was associated with increased odds of documentation across practice settings. This is consistent with findings from the World Mental Health Survey in which higher household income and educational level were associated with AUD persistence.^28^ Lower socioeconomic status predicts higher rates of alcohol-related morbidity and mortality,^18^ and higher income and socioeconomic status can shelter people from the consequences of AUDs. At the patient level, increased severity of alcohol-related problems among lower socioeconomic status groups can lead to easier diagnosis and higher likelihood of documentation.

Qualitative studies have further documented clinician-level barriers to screening that include stigma, shame, and discomfort; the presence of co-occurring behavioral health and chronic physical conditions increase difficulty with identifying alcohol-related problems.^7,8,29^ The lack of documentation does not necessarily mean that there was no clinical observation of an alcohol-related problem or counseling related to that problem. Clinicians require sufficient time and administrative support to fully document screening and intervention activities. When the US Veterans Administration changed its requirements to increase documentation of counseling, Berger et al^30^ documented that Veteran’s Administration clinicians were able to catch up on previously undocumented counseling events.

The study by Barrio et al^31^ of differential characteristics between patients with alcohol dependence receiving or not receiving treatment found that lower socioeconomic status, higher unemployment, older age, and a higher number of comorbidities predicted treatment. The analysis by Mukamal^32^ of data from the US Behavioral Risk Factor Surveillance System found systematic differences in self-reported receipt of alcohol counseling in primary care, with Black and Hispanic adults twice as likely to receive counseling compared with White adults. Data from the 2014-2016 US National Medical Ambulatory Care Survey reinforce disparities in alcohol screening, with screening for unhealthy alcohol use more likely if the patient was seen by their assigned primary care clinician, if they were a new patient, or if they had several chronic conditions.^14^

Findings regarding disparities in documentation are consistent with current literature investigating documentation of alcohol-related problems within primary care in the US and Europe. However, our findings move beyond prior studies reporting low rates of documentation because we modeled practice-level socioeconomic status in addition to individual-level demographic characteristics, self-reported alcohol risk behavior, and chronic conditions. Our large sample size and geographic variation allowed us to document statistically significant disparities in documentation that may inform efforts to improve universal screening for AUD and other substance use disorders in primary care.^33,34^

### Strengths and Limitations

The study results are likely generalizable to the US population of older adults with multiple chronic conditions who are engaged in primary care treatment. The IBH-PC practices were selected based on their capacity to integrate behavioral health services, which may be associated with a higher likelihood of identifying and documenting a substance use disorder.

This study has limitations. The population comprised adults engaged in care and diagnosed with multiple physical and behavioral conditions and is older than the general population at risk for AUD and associated morbidity and mortality. The study population does not include sufficient numbers to make valid inferences about racial or ethnic disparities in the likelihood of having a documented alcohol-related problem. Data were extracted from the IBH-PC trial, and data collection tools were not built to be directly comparable to standardized measures of excessive alcohol use, as defined by the US Centers for Disease Control and Prevention^26^ or the US National Survey on Drug Use and Health binge and heavy drinking indicators.^25^ The IBH-PC patient survey includes the GAIN-SS, but not items to specifically diagnose alcohol use disorder (*Diagnostic and Statistical Manual of Mental Disorders, Fifth Edition*^35^), as the US National Survey on Drug Use and Health does. Our indicator of higher-risk alcohol use does not specifically assess binge drinking as units of alcohol consumed on the same occasion; our measure of higher-risk drinking assesses the total number of drinks per day and per month only. Our measurement of higher-risk alcohol use is broad and does not include any indicator of craving, dependence, or withdrawal. The GAIN-SS is likely a more specific indicator of higher risk alcohol use than our constructed measure of higher-risk drinking. Both measures are associated with documentation in the multivariable models, suggesting that they are measuring different dimensions of higher-risk alcohol use.

The study’s data collection period included the start of the COVID-19 pandemic, when shelter-in-place policies were in effect and many health practices were only providing emergency care. During this time, assessment of alcohol-related problems may have been reduced differentially by SDI. However, all of the study participants were in care for multiple chronic conditions, and documentation of alcohol-related diagnoses in the EHR would not necessarily be associated with reduced screening at the practice level during the COVID-19 outbreak.

## Conclusion

The findings of this cross-sectional study reinforce the need to address practice-level barriers to documentation of alcohol-related problems in primary care. Low levels of documentation among participants who self-reported higher-risk drinking and scored positively on the GAIN-SS indicate a need for increased substance use screening, brief intervention, and access to specialty addiction treatment. Our finding that higher practice-level SDI is associated with reduced odds of documenting alcohol-related problems supports previous work citing clinical barriers, including training in and confidence using evidence-based tools, availability of appropriate screening tools, time, and staffing.^6,7,36,37,38^ Primary care practices in counties with higher levels of social deprivation may be more likely than practices in better-resourced counties to identify and document alcohol-related problems among their patients with multiple chronic conditions. At the same time, the multivariable model indicated higher odds of documentation among individuals with lower household incomes and worse health status, after adjusting for alcohol consumption. Future research including more detailed indicators of practice-level barriers to comprehensive documentation, including workforce limitations, patient panel size, and level of compensation from payers, may help to unpack the complex interactions between geography and practice resources.

## Notes

Supplement 1. **eTable 1.** Unadjusted Logistic Regressions—Odds of Higher Risk Alcohol Use Compared to Lower Risk or No Alcohol Use **eTable 2.** Odds of Documentation of Alcohol-Related Problems in the Electronic Health Record Excluding GAIN-SS and Alcohol Risk Categories as Covariates, Results of Multivariable Regression Analysis

Supplement 2.
Data Sharing Statement
