# Supplementary material for: Practice-Level Documentation of Alcohol-Related Problems in Primary Care
Source: JAMA Netw Open. 2023 Oct 19;6(10):e2338224. doi: 10.1001/jamanetworkopen.2023.38224 (PMC10587783; doi:10.1001/jamanetworkopen.2023.38224)
Supplement: Supplement 1. — eTable 1. Unadjusted Logistic Regressions—Odds of Higher Risk Alcohol Use Compared to Lower Risk or No Alcohol Use eTable 2. Odds of Documentation of Alcohol-Related Problems in the Electronic Health Record Excluding GAIN-SS and Alcohol Risk Categories as Covariates, Results of Multivariable Regression Analysis [file jamanetwopen-e2338224-s001.pdf]

Supplementary Online Content

Waddell EN, Leibowitz GS, Bonnelll LN, Rose GL, McGovern M, Littenberg B. Practice-level documentation of alcohol-related problems in primary care. *JAMA Netw Open*. 2023;6(10):e2338224. doi:10.1001/jamanetworkopen.2023.38224

eTable 1. Unadjusted Logistic Regressions—Odds of Higher Risk Alcohol Use Compared to Lower Risk or No Alcohol Use

eTable 2. Odds of Documentation of Alcohol-Related Problems in the Electronic Health Record Excluding GAIN-SS and Alcohol Risk Categories as Covariates, Results of Multivariable Regression Analysis

This supplementary material has been provided by the authors to give readers additional information about their work.

© 2023 Waddell EN et al. *JAMA Network Open.*

eTable 1. Unadjusted Logistic Regressions – Odds of higher risk alcohol use compared to lower risk or no alcohol use, N=3,105

**Unadjusted Logistic Regressions – Odds of higher risk alcohol use compared to lower risk or no alcohol use, N=3,105**		
	OR (95% CI)	*P*
**Demographic information and health status**		
Mean age ± SD, year	1.02 (1.01, 1.03)	<0.001
Mean age^2 ± SD, year	1.001 (1.0001, 1.005)	<0.001
Male sex	2.09 (1.64, 2.67)	<0.001
Female sex (ref)	--	--
Black race	0.46 (0.28, 0.75)	0.002
Other race, including not disclosed*	0.39 (0.23, 0.65)	<0.001
White race (ref)	--	--
Hispanic Ethnicity	0.22 (0.09, 0.54)	0.001
Low household income ($<30k)	0.42 (0.32, 0.55)	<0.001
College degree	1.37 (1.07, 1.75)	0.01
Urban Area defined by RUCA	1.03 (0.67, 1.22)	0.21
Number chronic conditions in electronic health record	0.93 (0.90, 1.03)	0.06
**Global Appraisal of Individual Needs – Short Screener (GAIN-SS)** ≥ 2 (Past Month)	15.83 (10.81, 23.19)	<0.001
**Practice-level Social Deprivation Index (SDI), units of 10**	0.94 (0.88, 0.99)	0.03

*Includes American Indian or Alaskan Native, Asian, Native Hawaiian/Other Pacific Islander, Other, Not disclosed

© 2023 Waddell EN et al. *JAMA Network Open.*

eTable 2. Odds of documentation of alcohol-related problems in the electronic health record excluding GAIN-SS and alcohol risk categories as covariates, results of multivariable regression analysis, N=2,944

	OR	95% CI	*P*
**Practice-level Social Deprivation Index (SDI)**, units of 10	0.88	0.80,0.98	0.02
**Demographic information and health status**			
Age	1.11	0.99,1.24	0.05
Age^2	0.998	0.997,0.999	0.015
Sex			
Male	2.31	1.65,3.25	<0.001
Female (ref)	--	--	--
Race			
Black	0.89	0.51,1.55	0.69
Other race, including not disclosed*	0.53	0.26,1.09	0.09
White (Ref)	--	--	--
Hispanic Ethnicity	0.82	0.34,1.95	0.66
Low household income ($<30k)	1.64	1.11,2.44	0.012
College degree	0.85	0.58,1.22	0.38
Urban Area defined by Rural Urban Commuting Area (RUCA)	0.97	0.89,1.05	0.49
Number chronic conditions in electronic health record	1.66	1.50,1.87	<0.001

*Includes American Indian or Alaskan Native, Asian, Native Hawaiian/Other Pacific Islander, Other, Not disclosed

© 2023 Waddell EN et al. *JAMA Network Open.*
