# Supplementary material for: Practice-Level Documentation of Alcohol-Related Problems in Primary Care
Source: JAMA Netw Open. 2023 Oct 19;6(10):e2338224. doi: 10.1001/jamanetworkopen.2023.38224 (PMC10587783; doi:10.1001/jamanetworkopen.2023.38224)
Supplement: Supplement 2. — Data Sharing Statement [file jamanetwopen-e2338224-s002.pdf]

Data Sharing Statement

Waddell. Practice-Level Documentation of Alcohol-Related Problems in Primary Care. *JAMA Netw Open*. Published October 18, 2023. doi:10.1001/jamanetworkopen.2023.38224

Data
Data available: No

Additional Information
Explanation for why data not available: The data will not be shared publicly due to the complexity of data use agreements with participating medical practices. Please contact the authors for access to a limited data set.
